# The Development and Validation of a Questionnaire to Investigate the Association Between Maternal Food Insecurity and Fetal Structural Anomalies: Delphi Procedure and Pilot Study

**DOI:** 10.1007/s10995-023-03675-8

**Published:** 2023-06-22

**Authors:** Drieda Zaçe, Ilda Hoxhaj, Tina Pasciuto, Maria Diakanthos, Flavia Beccia, Maria Luisa Di Pietro, Marco De Santis

**Affiliations:** 1 Section of Hygiene, University Department of Life Sciences and Public Health, Università Cattolica del Sacro Cuore, Roma, Italia; 2 Research Core Facility Data Collection G-STeP, Fondazione Policlinico Universitario Agostino Gemelli IRCCS, Roma, Italia; 3 Department of Woman and Child Health and Public Health – Public Health Area, Fondazione Policlinico Universitario A. Gemelli IRCCS, Roma, Italia

**Keywords:** Food insecurity, Insecurity, Women, Children, fetal structural anomalies, Questionnaire

## Abstract

**Introduction:**

Food insecurity represents a public health issue that has been associated with poor birth outcomes. We describe the methodological steps followed to structure and validate a questionnaire, which has the potential to contribute to the planning and conduction of future studies investigating the possible association between maternal food insecurity and fetal structural anomalies.

**Methods:**

We first conducted a literature review to structure and validate the questionnaire. Subsequently, we drafted the questionnaire based on the results of this review, further refined through two focus groups. Afterward, the questionnaire was submitted using the Delphi Method to a panel of experts for validation. We conducted a pilot study prior to recruiting the final sample.

**Results:**

The questionnaire consisted of sections covering information about socio-demographic characteristics, women’s health and lifestyle, pregnancy, and food security status. After the first Delphi round, the Content Validity Index (CVI) for each section ranged 0.81–0.85, while after the second round all items had a CVI of 1. The final version of the questionnaire, consisting of 87 items, was pilot tested among 20 participants. Cronbach’s Alpha for each section resulted in values higher than 0.6. The response rate ranged from 78 to 100%. A situation of food security was present in 85% of the participants, while 5% were in a situation of mild food insecurity and 10% of moderate food insecurity.

**Conclusion:**

The questionnaire has appropriate measurement properties, and is an adequate instrument to evaluate the association between maternal food insecurity and fetal structural anomalies.

**Supplementary Information:**

The online version contains supplementary material available at 10.1007/s10995-023-03675-8.

## Significance

***What is already known?*** Food insecurity in pregnant women is a public health concern, since it affects both the woman and her child. This condition leads to poor health outcomes, such as gestational hypertension, preeclampsia, gestational diabetes, and anemia for the woman, and low birth weight, shorter gestational age, and chronic diseases in adulthood for the child. However, to date, few studies have investigated the potential association between food insecurity in women during pregnancy and fetal structural anomalies.

***What this study adds?*** To our knowledge, in Europe this is the first study to evaluate the association of interest. Understanding and assessing the potential association of food insecurity in pregnant women and fetal structural anomalies might help policymakers design and implement policies, interventions, and screening strategies to mitigate food insecurity in pregnant women. In this regard, there is the need for validated instruments, and through this study we provide the detailed methodology process in developing and structuring a questionnaire that might be used as a guide tool for future studies. Identifying women at risk of food insecurity and assisting them might help in preventing poor health outcomes and fetal structural anomalies, which could also decrease the burden on health systems.

**Supplementary Information:**

The online version contains supplementary material available at 10.1007/s10995-023-03675-8.

## Introduction

Factors such as economic downturns, conflicts, climate change, and pandemics are threatening the global food security status. Food insecurity - as assessed by United States Department of Agriculture - is an economic and social condition, at an individual and household level, of limited or uncertain access to adequate food (Alisha Coleman-Jensen et al., 2020).

The Food and Agriculture Organization (FAO) has estimated that globally the number of people affected by hunger has been rising since 2014. Projections show that the world is not on track to achieve Zero Hunger by 2030 and despite some progress, most indicators are also not on track to meet global nutrition targets (FAO, IFAD, UFAO, IFAD, UNICEF, WFP and WHONICEF, 2020).

While poverty and low income are among the most important determinants associated with food insecurity, adequate household income alone is not sufficient to ensure food security (Ivers & Cullen, 2011). Specific population groups may be more vulnerable to negative effects associated with food insecurity and their situation is likely to further deteriorate due to the health and socio-economic impacts of the COVID-19 pandemic (FAO, IFAD, UFAO, IFAD, UNICEF, WFP and WHONICEF, 2020).

Pregnant women represent one of the vulnerable population groups and are at risk for several negative health outcomes. Food insecurity has a double burden, leading to consequences for the woman herself and her child (Laraia et al., 2006). Food insecurity might have particular importance for women during pregnancy because of three main reasons: nutrient demands are higher, the effort required for food preparation may be more difficult, and pregnant women may be obliged to leave the workforce, especially in later pregnancy, which leads to financial strain (Laraia et al., 2006).

Pregnant women who experience household food insecurity also may be at greater risk of pregnancy complications (Laraia BA., Leung CW., Atkins V, 2013). Food insecurity has been associated with low birth weight due to the presence of chronic stressors, including having difficulty obtaining food, and gestational diabetes, with implications for both the fetus (e.g., macrosomia) and the mother (e.g., a significant precursor to type II diabetes later in life) (Borders et al., 2007; Laraia BA., Leung CW., Atkins V, 2013).

Furthermore, there is a potential correlation between food insecurity in women during pregnancy and fetal structural anomalies, however few studies have investigated it. A case-control study, conducted in the United States, hypothesized that food insecurity may increase risks of birth defects, because it is an indicator of increased stress or compromised nutrition (Carmichael et al., 2007). This study showed that maternal food insecurity was associated with increased risk of certain birth defects, such as anencephaly, cleft palate, d-transposition of the great arteries, tetralogy of Fallot, spina bifida (Carmichael et al., 2007).

In Europe no studies have investigated the possible correlation between food insecurity in pregnant women and fetal structural anomalies. To fill this literature gap, a multicenter case-control study was planned in Italy (Zaçe et al., 2020). This ongoing study aims to investigate the impact of food insecurity among pregnant women before and during pregnancy on fetal structural anomalies.

To collect the information that could answer this objective, we developed and validated an instrument (questionnaire) which consists of four sections: (1) Socio demographic information; (2) Woman’s health status; (3) Household food security assessment; (4) Pregnancy information.

This paper describes the methodological steps followed to structure and validate the questionnaire, which has the potential to contribute to the planning and conduction of future studies investigating the possible association between maternal food insecurity and fetal structural anomalies.

## Materials and Methods

**Fig. 1:**
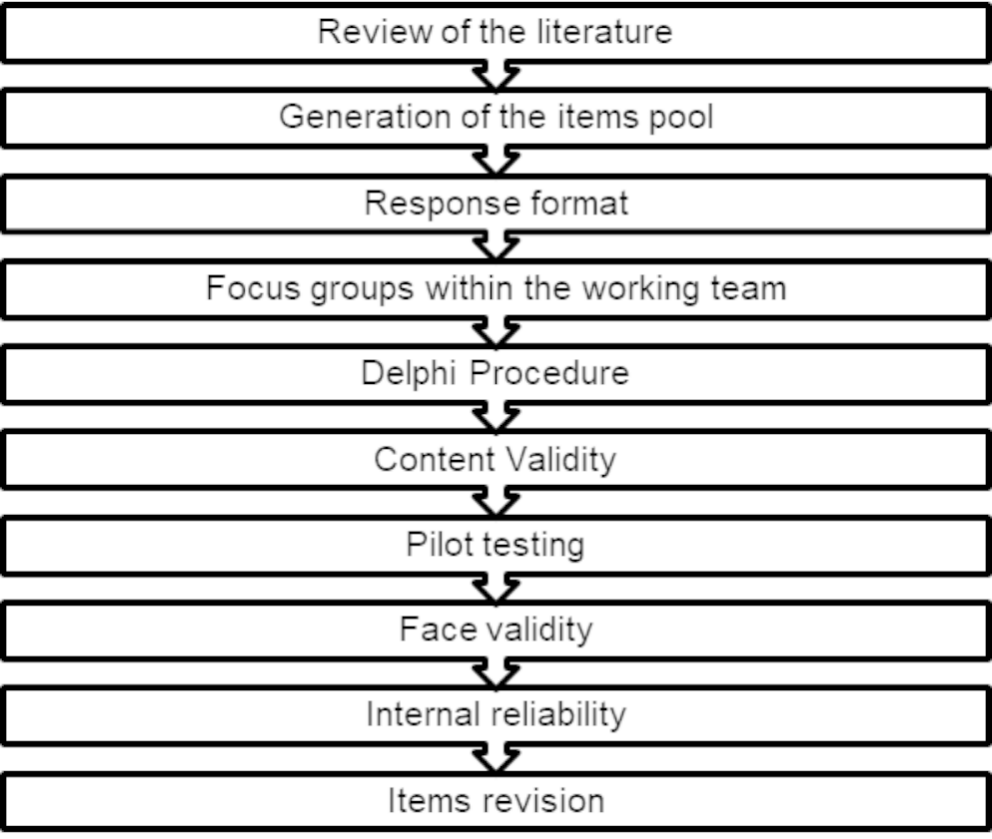
Flowchart of the construction and validation process

The structuring and validation of the questionnaire was carried out in different stages (Fig. 1) and lasted ten months. The first stage consisted of an extensive literature review to collect the available information in order to structure the questionnaire. Based on the results of the literature review, a first draft of the questionnaire was developed and further refined through two focus groups organized within the research team. Afterwards, the questionnaire was submitted to a panel of experts for validation, through a Delphi method. Prior to recruiting the final sample, a pilot study was conducted, in which the questionnaire was answered by a small sample of the target population.

### Step 1: Review of the Literature and Items Generation

The structuring of the questionnaire was based on a literature review which had two aims: (1) to identify any existing tools that had assessed the possible association between fetal structural anomalies and food insecurity, and (2) to create a pool of items that could be useful in the construction of the questionnaire.

A search string using the terms “food security”, “food insecurity”, “food access”, “malformations”, fetal structural anomalies”, and “congenital abnormalities” was built. PubMed and Scopus electronic databases were searched, without any time limits, to retrieve potential eligible articles, published until October 10th 2018.

The data extracted from the included articles were pooled into a first version of the questionnaire, which was evaluated through a focus group methodology. Three public health researchers, a statistician, and a gynaecologist participated in the focus group.

#### Definition of Food Security and Association With Structural Malformations

Food security/insecurity was measured using the validated Household Food Insecurity Access Scale (HFIAS) (Coates et al., 2007). Multinomial logistic regression models were performed to assess the impact of food insecurity on fetal structural anomalies. Based on the previously published literature, several variables will be considered as covariates, including mother’s age, gestational age, race/ethnicity (White; Hispanic; Asian; African; Mixed; Other), education (Elementary school; High school; University; Post-university), occupation, household income, body mass index before pregnancy (kilograms per square meter), alcohol (frequency, type and quantity of alcohol at the moment and at least 1 month before conception), smoking (frequency and number of cigarettes at the moment and at least 3 months before conception), intake of folic acid or other vitamins supplement, and dietary habits in terms of quantity and frequency of consuming fish, meat, fruits, vegetables, and dairy, specific diet and changes in the diet.

The response format included: dichotomous answers (yes/no), multiple choice questions and 3 points Likert scale (rarely, sometimes, always). The questionnaire was designed to be self-administered.

### Step 2: Delphi Procedure

#### Delphi Method

The questionnaire designed by the internal focus group, was further validated through the Delphi method. The Delphi method is a well-suited technique for consensus-building by using a series of questionnaires to collect data from a panel of selected subjects on a particular topic (Hsu & Sandford, 2007; Schmalz et al., 2021).

The main characteristics are: (1) anonymity - the experts do not know the responses of the other specialists; (2) feedback – the experts can suggest some additional information for research or justify their choices; (3) iterations - the number of rounds; and (4) statistical analysis (Trakman, Forsyth, Hoye, & Belski, 2017).

#### Selection of the Experts’ Panel and Recruitment

The criteria for selecting the Delphi panel of experts included at least one of the following: (1) publications on the topic of fetal structural anomalies or food insecurity; (2) experience and involvement in working with pregnant women; (3) knowledge and expertise regarding pregnancy risk factors for fetal structural anomalies; and (4) expertise in the field of paediatrics and food insecurity. Since the Delphi method does not rely on a strictly defined number of experts (Glenn & Gordon, 2009), we decided to enroll six experts in different fields such as gynaecology, paediatrics, public health, bioethics and statistics. All identified experts were individually contacted via e-mail and were asked for their availability to be part of the Delphi procedure.

#### Data Collection

After the invited experts agreed to participate, an e-mail was sent to each of them, including the questionnaire and information for the validation process. Experts were invited to review the content of the items and to rate each one in terms of its validity and relevance by indicating a value from 1 to 5 on a Likert scale, where 1 = strongly disagree (this question should not be included in this questionnaire/it is irrelevant) and 5 = strongly agree (this question is relevant and should be included in the questionnaire). Furthermore, experts were given the possibility to list any additional comments or questions. At the end of the first round, the questionnaire was modified integrating feedback from all participants and sent again to the panel for a Delphi second round.

#### Content Validity

Content validity determines the ability of the selected items to reflect the variables of the construct in the measure. It addresses the degree to which the items of an instrument sufficiently represent the content domain. It can be quantified through the Content Validity Index (CVI), which derives from the ratio between number of experts that rate a singular item with 4 and 5 (maximum rates) and the total number of experts involved. CVI has a range from 0 to 1, or from 0 to 100%. A CVI greater than 79% is deemed to be suggestive of the item’s insertion in the questionnaire, a rate between 70 and 79% is considered indicative for the revision of the item and a rate lower than 70% is deemed to be suggestive of removing the item (Zamanzadeh et al., 2015).

### Step 3: Pilot Study

#### Characteristics of the Pilot Study

The final version of the questionnaire, established after the Delphi method, was pilot tested to assess its reliability and to identify any lack of clarity before implementing a wider administration.

The pilot study was conducted at the Obstetrics and Obstetrical Pathologies Unit of the Coordinating Center, between October and December 2019. A convenience sample was enrolled, taking into consideration references stating that a sample of < 15 units is not recommended in the pre-test (Perneger et al., 2015).

In the pilot test were included as cases pregnant women, 18 years or older, with a positive result for a fetal structural anomaly, in a prenatal ultrasound examination during the II–III trimester of gestation. The controls were pregnant women, 18 years or older, with a negative result for a fetal structural anomaly, in a prenatal ultrasound examination during the II–III trimester of gestation. Pregnant women with any of the following conditions were excluded: (1) fetuses diagnosed with chromosomal anomalies: (2) twins or more pregnancies, (3) uterine malformations, (4) suspected specific syndromes, (5) exposure to known teratogens, and (6) any type of fetal functional anomaly. Detailed information on the methodology of the case-control multi-center study is published elsewhere (Zaçe et al., 2020).

#### Face Validity

The questionnaire was delivered to each participant by a member of the research team, who asked feedback on the comprehensiveness and clarity of the questions. After completing the questionnaire, each woman was asked about her experience during the completion, whether there were confusing questions or non-comprehensive language, and about any recommendations to improve the questionnaire. Responses were gathered and analyzed to make necessary changes to the terminology and wording used.

#### Internal Reliability

Reliability is the measurement of the correlations among the items that form part of an instrument or internal consistency, described as the extent to which all the items in a test measure the same concept. Hence, it is connected to the inter-relatedness of the items within the test. The reliability of an instrument can be quantified through Cronbach’s Alpha, which is expressed as a number between 0 and 1. A value > 0.80 is considered to be almost perfect, from 0.80 to 0.61 substantial, from 0.60 to 0.41 moderate and < 0.41 poor (Streiner, 2003; Tavakol & Dennick, 2011).

## Results

### Structuring of the Questionnaire

The questions were elaborated based on the literature review, which identified only two case-control studies published in this topic, in 2007 and 2016. In particular, these studies evaluated the association of maternal food insecurity with the risk of birth defects in the United States of America (Carmichael et al., 2007), and the risk of oral clefts in Thailand (McKinney et al., 2016). Based on the retrieved data (after the internal focus group), it was deemed important to include information on socio-economic variables, woman’s health status, pregnancy, and food security status. The elaborated questionnaire consisted of 86 questions, organized in four sections, as follows:

1. personal information (8 questions).
2. woman’s health (39 questions).
3. food security status (18 questions).
4. information about the pregnancy (21 questions).

### Delphi Procedure

#### First Delphi Round

As shown in Table 1, the mean experts’ evaluation obtained for each item of the questionnaire ranged from 3.33 to 5, out of a maximum of 5. The questions with the highest score (mean value = 5), were those related to folic acid intake and smoking. The questions with the lowest score (mean value < 4) were related to the presence of chronic diseases, exposure to ionizing radiation, and coffee consumption, which were further revised. CVI for each item ranged from 0.5 to 1.0, indicating the need for modifications of questions with a CVI lower than 0.8 (Table 1).

**Table 1:** Context validity index of each item of the questionnaire, after the first round of Delphi, assessed by the six experts

Sections	Question	Expert							Mean	CVI	
		1	2	3	4	5	6				
Personal information	1. Age	5	5	3	5	5	5		4.67	0.83	
	2. Ethnicity	5	5	3	4	4	4		4.17	0.83	
	3. Civil Status	5	5	3	4	5	5		4.50	0.83	
	4. Duration of living in Italy	5	5	3	5	5	5		4.67	0.83	
	5. Education level	5	5	3	5	5	5		4.67	0.83	
	6. Paying job	5	5	3	5	5	5		4.67	0.83	
	7. Working sector	3	5	3	5	4	4		4.00	0.67	
	8. Income	5	3	3	5	5	5		4.33	0.83	
									**4.46**	**0.81**	
Woman’s health status	1. Height (cm)	5	5	3	5	5	5		4.67	0.83	
	2. Weight (kg)	5	5	3	5	5	5		4.67	0.83	
	3. History of chronic diseases	1	5	3	5	5	4		3.83	0.67	
	4. Type of disease (specify)	5	5	3	5	4	4		4.33	0.83	
	5. Use of medication for chronic diseases	1	5	3	5	5	5		4.00	0.67	
	6.Type of medication (specify)	5	5	3	5	5	5		4.67	0.83	
	7. Exposure to ionizing radiations	5	4	4	4	3	3		3.83	0.67	
	7a. Age at exposure to ionizing radiations	5	3	3	5	5	5		4.33	0.67	
	9. Age at first menstrual cycle	5	5	3	5	5	5		4.67	0.83	
	10. Use of medication for menstrual regulation	5	5	3	5	4	5		4.50	0.83	
	10a. Type of medication taken	5	5	3	5	5	5		4.67	0.83	
	11. Family history of congenital malformation	5	5	4	4	4	5		4.50	1.00	
	12. Smoking (habit) Yes/No	1	5	3	5	5	5		4.00	0.67	
	12a. Age at which smoking habit has started	1	5	3	5	5	5		4.00	0.67	
	12b. Number of cigarettes per day	5	5	3	5	5	5		4.67	0.83	
	12c. Ever smoked	5	5	5	5	5	5		5.00	1.00	
	12d. Time that stopped smoking	5	5	4	5	5	5		4.83	1.00	
	13. Alcohol consumption	5	5	3	5	5	5		4.67	0.83	
	13a. Alcohol consumption frequency	5	5	4	5	4	5		4.83	1.00	
	13b. Type of alcohol	5	5	3	5	4	4		4.00	0.83	
	13c. Age at which alcohol consumption started	5	5	3	4	5	5		4.50	0.83	
	13d. Age at which alcohol consumption stopped	5	5	3	4	5	5		4.50	0.83	
	14. Coffee consumption (not decaffeinated)	5	5	3	5	5	5		4.67	0.83	
	14a. Number of coffees per day	5	5	3	5	3	5		4.33	0.67	
	14b. Age at which coffee consumption started	5	5	3	5	5	5		4.67	0.83	
	14c. Age at which coffee consumption ended	5	5	3	5	5	5		4.67	0.83	
	14d. Coffee consumption (other types)	1	5	1	5	5	3		3.33	0.5	
	15. Drug use	5	5	3	4	5	5		4.50	0.83	
	15a. Age at which drug use started	5	5	3	5	5	5		4.67	0.83	
	15b. Drug use currently	5	5	3	5	5	5		4.67	0.83	
	15c. End of drug consumption	5	5	3	5	5	5		4.67	0.83	
	16. Specific diets	5	5	3	5	4	5		4.50	0.83	
	16a. Daily meals	5	5	3	5	5	5		4.67	0.83	
	16b. Changes in nutritional habits	5	5	3	5	5	5		4.67	0.83	
	16c. Meat consumption	5	5	3	5	5	5		4.67	0.83	
	16d. Fish consumption	5	5	3	5	5	5		4.67	0.83	
	16e. Vegetable consumption	5	5	3	5	5	5		4.67	0.83	
	16 f. Fruit consumption	5	5	3	5	5	5		4.67	0.83	
	16 g. Dairy products consumption	5	5	3	5	5	5		4.67	0.83	
									**4.48**	**0.82**	
Food security status	1. Worries for not getting enough food	5	5	4	5	5	5		4.83	0.83	
	1a. How often has it happened (point 1)	5	5	4	5	5	5		4.83	0.83	
	2. Can’t eat preferred foods due to lack of resources	5	5	3	4	5	5	4.50		0.83	
	2a. How often has it happened? (point 2)	5	5	3	5	5	5	4.67		0.83	
	3. Can’t eat often a variety of foods due to scarce resource	5	5	3	4	5	5	4.50		0.83	
	3a. How often has it happened? (point 3)	5	5	3	5	5	5	4.67		0.83	
	4. Eating foods that didn’t like due to lack of resources	5	5	3	4	5	5	4.50		0.83	
	4a. How often has it happened? (point 4)	5	5	3	5	5	5	4.67		0.83	
	5. Eating less food due to scarce resources	5	5	3	4	5	4	4.33		0.83	
	5a. How often has it happened? (point 5)	5	5	3	5	5	5	4.67		0.83	
	6. Skipping a meal due to scarce resources	5	5	3	4	5	5	4.50		0.83	
	6a. How often has it happened? (point 6)	5	5	3	5	5	5	4.67		0.83	
	7. Lack of food due to lack of resources	5	5	3	4	5	5	4.50		0.83	
	7a. How often has it happened? (point 7)	5	5	3	5	5	5	4.67		0.83	
	8. Going to sleep hungry due to lack of food	5	5	3	5	5	5	4.67		0.83	
	8a. How often has it happened? (point 8)	5	5	3	5	5	5	4.67		0.83	
	9. A whole day or night without eating due to lack of resources	5	5	3	5	5	5	4.67		0.83	
	9a. How often has it happened? (point 9)	5	5	2	5	5	5	4.50		0.83	
								**4.61**		**0.85**	
Pregnancy Information	1. Gestational age	5	5	3	5	5	5	4.67		0.83	
	2. Current intake of folic acid	5	5	3	5	5	5	4.67		0.83	
	2a. When has the intake of folic acid started	5	5	5	5	5	5	5.00		1.0	
	3. Vitamins intake	5	5	3	5	5	5	4.67		0.83	
	3a. Type of vitamins	5	5	3	5	5	5	4.67		0.83	
	3b. When has the vitamins’ intake started	5	5	3	5	5	5	4.67		0.83	
	4. Chemical substances exposure during pregnancy	5	5	2	4	5	4	4.17		0.83	
	4a. Type of chemical substances	5	5	3	4	5	4	4.33		0.83	
	5. Current diseases	5	5	3	5	5	5	4.67		0.83	
	5a. Type of diseases	5	5	3	5	5	5	4.67		0.83	
	6. Infections during pregnancy	5	5	3	5	5	5	4.67		0.83	
	6a. Type of infections	5	5	3	5	5	5	4.67		0.83	
	7. Use of artificial reproduction technology	5	5	4	5	5	5	4.83		0.83	
	7a. Type of techniques	5	5	3	5	5	5	4.67		0.83	
	8. Current intake of medications	5	5	3	5	5	5	4.67		0.83	
	8a. Type of medications	5	5	3	5	5	5	4.67		0.83	
	9. Number of previous pregnancies	5	5	3	5	5	5	4.67		0.83	
	10. Number of prior natural births	5	5	3	5	5	5	4.67		0.83	
	11. Number of prior caesarean sections	5	5	3	5	5	5	4.67		0.83	
	12. Number of stillbirths	5	5	3	5	5	5	4.67		0.83	
	13. Number of miscarriages	5	5	3	5	4	5	4.50		0.83	
								**4.64**		**0.85**	

Based on the experts’ suggestions received in the first round, additional changes were implemented in each section, as follows:

*In the first section were*:

- Added four questions concerning: the type and environment of woman’s’ employment (1), family size (1), working sector (1), and type of employment of the child’s father (1).
- Modified: two questions on ethnicity (1), civil status (1) and type of employment (1).

*In the second section were*:

- Added four questions on: weight before pregnancy (1), number of cigarettes smoked before quitting (1), amount of fish consumed (1), and frequency of drug use during pregnancy (1).
- Modified nine question on: smoking (2), exposure to ionizing radiations (1), chronic diseases (1), medications (2), age at first drug use (1), diet (1), and coffee consumption (1).
- Removed two questions regarding: the age at ionizing radiation exposure (1) and use of other types of coffee (1).

*In the fourth section were*:

- Added three questions about: the sex of the fetus (1), urinary infections (1) and genital infections (1).
- Modified five questions on: vitamins assumptions (1), medications taken unconsciously during pregnancy (1), folic acid assumption (1), chemical compound exposure (1) and chronic diseases (1).

**Fig. 2:**
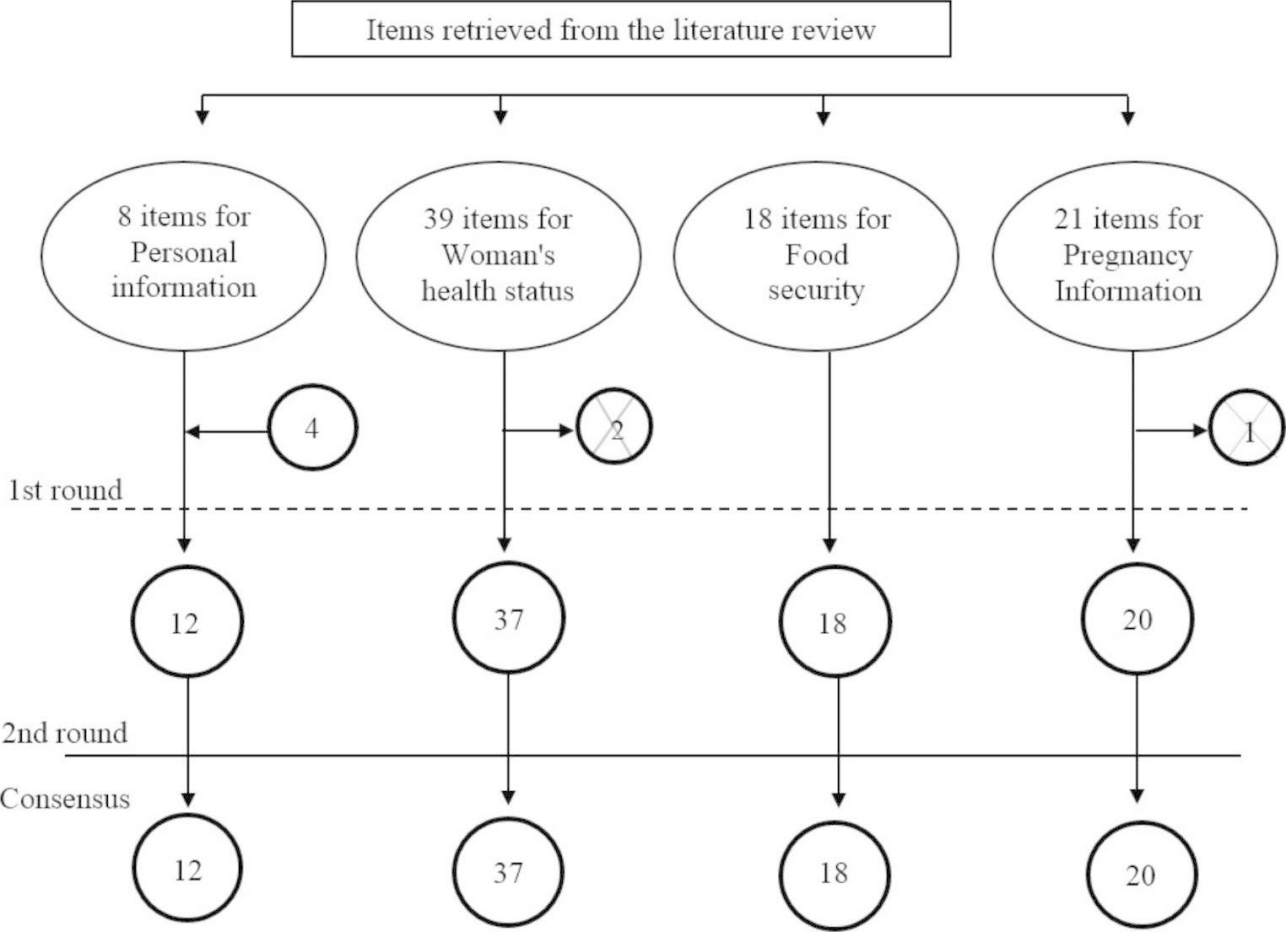
Flowchart of the structuring of the questionnaire with respective number of questions per section

After these modifications, the questionnaire consisted of a total number of 87 questions (Fig. 2) and was submitted to a second Delphi round.

#### Second Delphi Round

After the second Delphi round, a few changes were suggested, such as providing examples about ionizing radiations and chemical compounds exposure and specifying the variable “economic resources” at the question on food insecurity. The inter-agreement rate among experts was 100% and CVI was 1 for all identified items, obtaining the consensus in this round. The final version of the questionnaire contained 87 questions: 12 on personal information; 37 on woman’s health status; 18 on food security status; and 20 on pregnancy information section (Fig. 2).

### Pilot Study

The questionnaire was pilot-tested in a sample of 20 pregnant women, of which six cases and 14 controls. The fetal structural anomalies diagnosed by ultrasound examination were megabladder (16.6%), omphalocele (16.6%), cardiac malformation (50.2%) and renal malformation - stenosis of the joint (16.6%).

#### Cronbach’s Alpha and Face Validity

Regarding the reliability of the questionnaire, Cronbach’s Alpha obtained, for each section resulted as following:

1. “Personal information”: 0.607.
2. “Woman’s health status”: 0.658.
3. “Food security status”: 0.639.
4. “Pregnancy Information”: 0.600.

A Cronbach’s Alpha higher than 0.6, for each section, indicated a substantial internal reliability of the questionnaire. None of the women asked to participate to the study refused. The mean time of compilation for the questionnaire was 15 min. The response rate of the questions ranged from 78 to 100%. Questions related to alcohol and drugs consumption had the lowest response rate. Women were asked to end the questionnaire in case of a negative answer at the question regarding previous pregnancies. Participants reported that the questionnaire was long but comprehensive of different relevant issues and did not provide any further suggestions.

#### Description of the Pilot Sample

The mean age of the women enrolled in the pilot study was 34 years old. About 84% were white, 78% had an Italian nationality and 35% possessed a bachelor degree. Almost all the women had a household composed of two or three members; 70% were married and 25% lived with their partner. Only 45% of participants had a full-time job and in 46% the annual income was higher than 35,000 euros (Table 2).

**Table 2:** Characteristics of the women included in the pilot study

Characteristics	Number (%)
Mean Age 34 (18–43) years old	
**Ethnicity** ^a^	
White	16/19 (84.0)
Other	3/19 (16.0)
**Marital status**	
Married	14 (70.0)
Single	1 (5.0)
Cohabiting	5 (25.0)
**Educational level**	
Elementary school	3 (15.0)
High school	7 (35.0)
University degree	7 (35.0)
Post-university	3 (15.0)
**Occupation**	
No	6 (30.0)
Yes, full time	9 (45.0)
Yes, part time	5 (25.0)
**Household’s members** ^a^	
2	9/19 (47.4)
3	9/19 (47.4)
4	1/19 (5.2)
**Household’s income ‡**	
< 10,000€	1/15 (6.7)
10,000–14,999€	0 (0)
15,000–19,999€	3/15 (20.0)
20,000–24,999€	0 (0)
25,000–29,999€	3/15 (20.0)
30,000–34,999	1/15 (6.7)
> 35,000€	7/15 (46.7)

^a^ Information available for 19/20 women. ‡ Information available for 15/20 women

#### Information on Women’s Health and Pregnancy

The mean body weight during pregnancy was 66 kg (min. 45 kg; max. 95 kg), whereas before pregnancy was 61 kg (min.43 kg; max. 94 kg) (Supplementary File 1).

The chronic diseases encountered were anemia, hypothyroidism / hyperthyroidism, coagulopathies, and anxiety, and the most used medications were cardiospirina, clexane, deltacortene, eutirox, eviplera and tirosint. The most used medications for the regulation of the menstrual cycle were Diana and Jasmine. 15% were current smokers and had started at age of 13, 14 or 22 years old, smoking 3–4 cigarettes/day. Half of the woman who had stopped smoking, did so before conception or when they knew about the pregnancy. Most of them had a balanced diet, consuming an adequate amount of fish, meat, fruits and vegetables. Women in the sample had a gestational age between 16 and 37 weeks (Table 6). Most of them had started taking folic acid before conception.

#### Data Regarding food Insecurity in the Sample

A situation of food security was present in 85% of the participants, while 5% were in a situation of mild food insecurity and 10% of moderate food insecurity. None of the women were in a situation of severe food insecurity. Following a dichotomous variable, it is concluded that 85% of women were in a situation of food security, whereas 15% were experiencing food insecurity at some level. Food insecurity was present in 16% of cases and 14% of controls.

## Discussion

Food insecurity is a public health issue and it has been associated with several negative health outcomes, affecting especially vulnerable individuals, such as children and pregnant women (Ivers & Cullen, 2011; Moafi et al., 2018). Available publications have shown how inadequate or poor nutrition in pregnancy is linked to the manifestation of problems in the newborn, such as low birth weight, low gestational age, and a greater risk in the onset of diseases in the adult life (Grilo et al., 2015). Several studies have reported an association between maternal food insecurity and fetal structural anomalies (McKinney et al., 2016), but there is not enough data to draw conclusions.

Given the gap in evidence and the emerged need for further evalutaions our study presenting the process of developing and validating a questionnaire, is the first methodological phase of a multi-center case-control study that aims to evaluate the association of interest (Zaçe et al., 2020). The final version of the questionnaire comprised 87 items, with an estimated completion time of 15 min.

The questionnaire was developed based on a literature review and focus group, and then defined through a rigorous multidisciplinary Delphi method involving experts in the field. This method allowed us to modify the elaborated questions identified through the literature review, according to the experts’ suggestions, hence enriching the contents with all the useful elements that could help in understanding the association of interest. Although there is not a consensus on methodology of the preparatory phase, structuring the questionnaire based on a literature review has been considered an approach of quality.

The high consensus rate since the first Delphi round shows that the literature review and subsequently the focus group conducted were of substantial help in structuring the questionnaire. The consensus rate of 100% at the second round was high, indicating the validity and quality of the questionnaire (Jünger, Payne, Brine, Radbruch, & Brearley, 2017). The panel was composed of six experts, following the suggestion of Steiner and Norman who state that the panel should include 3–10 experts (“Health Measurement Scales: A practical guide to their development and use - Oxford Medicine,” n.d.). The anonymity reduced the possibility that the experts could be influenced by each other’s opinion (Bhandari & Hallowell, 2021).

Content validity, face validity, and internal reliability of the questionnaire resulted being above acceptable values, which ascertains the methodological strength of the questionnaire (Trakman et al., 2017). The CVI was higher than 0.8 for each section of the questionnaire, which indicates that the included items were able to address our objectives and sufficiently represented the content domain (Zamanzadeh et al., 2015).

The high response rate in the pilot study showed that the length of the questionnaire did not impact completion. Although there is not evidence on mean time of completion, it has been suggested that the length of a questionnaire does not affect the quality of responses (Iglesias & Torgerson, 2000). The structure and the language used in the questions’ formulation were likely fully understood, offering the informative content that the researchers were expecting.

The reliability of the questionnaire was concluded to be substantial, considering the values of Cronbach’s Alpha higher than 0.6. The small size of the sample used to conduct the pilot study may have played an important role in obtaining these values. Increasing the sample, we expect to have higher Cronbach’s Alpha values. However, these substantial values confirmed that the sections that structure the questionnaire allow testing the potential association between gestational food insecurity and fetal anomalies.

The pilot study had as its main objective the verification of the comprehensibility, the completion time, the attitude of the participants, and the reliability of the questionnaire. The objective was not to test the possible association between food insecurity and fetal structural anomalies. This objective will be achieved by the broad application of the questionnaire, which based on the results presented in this work, has a satisfactory validity and reliability. The sample of this pilot study reflected the population of interest based on the characteristics of the participants. Furthermore, the prevalence of food insecurity in the sample was comparable to the one reported by a study conducted among Italian households with children (15.0% vs. 14.5%) (Zaҫe, Di Pietro, Reali, de Waure, & Ricciardi, 2021).

### Limitations and Strengths

Regarding the limitations of this study, the questionnaire has been elaborated based on the literature review that included only articles published in English, thus indicating the potential presence of publication bias. During the pilot study, social desirability may have biased women’s responses. It has been reported that parents may be ashamed of their inability to feed their family and their children, which could result in under-reporting of the prevalence of food insecurity and misclassification of the individuals in the respective food insecure and food secure categories (Fram et al., 2013). In addition to this, it is possible that response bias has also occurred due to the self-reported responses provided.

The major strength of this study derives from the method used for the development and validation of the questionnaire which, through the Delphi procedure adopted here, allowed for improvement of the potential performance of the questionnaire.

To the best of our knowledge this is the first questionnaire developed and validated in Europe which has the potential to investigate the association between maternal food insecurity and fetal structural anomalies and could be used in many different settings. The questionnaire was clearly understood by the target population, showing good face validity and reliability, being concise and relevant. The questionnaire has adequate measurement properties, being established as an adequate instrument to evaluate the association between maternal food insecurity and fetal structural anomalies. In addition, an already validated and recognized scale was used to assess food insecurity among women.

Since there are only a few similar studies that describe a possible association between food insecurity and fetal structural anomalies, the data deriving from the compilation of this questionnaire could help to fill this gap in literature, thus acting as a methodological basis for future research in other countries. The research in this field could be enhanced by the availability of a validated questionnaire, able to investigate this association. It could guide stakeholders and policymakers to develop and implement policies and interventions that could tackle food insecurity, positively influencing on the health of women and their children.

## Electronic Supplementary Material

Below is the link to the electronic supplementary material.

Supplementary Material 1

Supplementary Material 2

## Data Availability

All data generated or analysed in this study are included in the article and its supplementary files.

## Code Availability

(Software application or custom code) Not applicable.
