# Supplementary material for: The Development and Validation of a Questionnaire to Investigate the Association Between Maternal Food Insecurity and Fetal Structural Anomalies: Delphi Procedure and Pilot Study
Source: Matern Child Health J. 2023 Jun 22;27(9):1518–28. doi: 10.1007/s10995-023-03675-8 (PMC10359368; doi:10.1007/s10995-023-03675-8)
Supplement: Supplementary file 1 — Supplementary Material 1 [file 10995_2023_3675_MOESM1_ESM.docx]

**Supplementary File 1.** Women’s health and lifestyle characteristics

| **Variable** | **Number (%)** |
| --- | --- |
| **Mean height; cm (range)** | 162.9 (153-172) |
| **Mean weight; kg (range)** | 66.6 (45-95) |
| **Mean weight before pregnancy; kg (range)** | 61.8 (43-94) |
| **History of chronic diseases** |  |
| No | 13 (65.0) |
| Yes | 7 (35.0) |
| **Use of medications*** |  |
| No | 8/19 (42.1) |
| Yes | 11/19 (57.9) |
| **Exposure to radiation** |  |
| No | 15 (75.0) |
| Yes | 5 (25.0) |
| **Mean Age menstrual cycle (years)** | 12.6 (10-15) |
| **Medications for menstrual cycle *** |  |
| No | 13/19 (68.4) |
| Yes | 6/19 (31.6) |
| **Family congenital malformations *** |  |
| No | 12/19 (63.2) |
| Yes | 1/19 (5.3) |
| I don't know | 6/19 (31.5) |
| **Smoking at the moment** |  |
| No | 17 (85.0) |
| Yes | 3 (15.0) |
| **Ever smoked ‡** |  |
| No | 6/13 (46.2) |
| Yes | 7/13 (53.8) |
| **Alcohol consumption during pregnancy** |  |
| No | 19 (95.0) |
| Yes | 1 (1.0) |
| **Coffee consumption during pregnancy** |  |
| No | 9 (45.0) |
| Yes | 11 (55.0) |
| **Ever used drugs *** |  |
| No | 18/19 (94.7) |
| Yes | 1/19 (5.3) |
| **Specific diet *** |  |
| No | 15/19 (78.9) |
| Vegetarian | 1/19 (5.2) |
| Intolerance | 1/19 (5.2) |
| Hypocaloric | 2/19 (10.4) |
| **Daily meals** |  |
| <3 | 1 (5.0) |
| 3 or 4 | 15 (75.0) |
| 5 | 4 (20.0) |
| **Changes in nutritional habits *** |  |
| No | 12/19 (63.2) |
| Yes, quantitative improvement | 4/19 (21.0) |
| Yes, qualitative improvement | 2/19 (10.5) |
| Yes, quantitative worsening | 1/19 (5.3) |
| Yes, qualitative worsening | 0 |

* Information available for 19/20 women. ‡ Information available for 13/20 women.
