# Supplementary material for: The Development and Validation of a Questionnaire to Investigate the Association Between Maternal Food Insecurity and Fetal Structural Anomalies: Delphi Procedure and Pilot Study
Source: Matern Child Health J. 2023 Jun 22;27(9):1518–28. doi: 10.1007/s10995-023-03675-8 (PMC10359368; doi:10.1007/s10995-023-03675-8)
Supplement: Supplementary file 2 — Supplementary Material 2 [file 10995_2023_3675_MOESM2_ESM.docx]

**Supplementary File 2.** Pregnancy-related information

| **Variable** | **Number (%)** |
| --- | --- |
| **Sex of the fetus** ‡ |  |
| Female | 9/17 (52.9) |
| Male | 8/17 (47.1) |
| **Folic acid intake at the moment** |  |
| No | 3 (15.0) |
| Yes | 17 (85.0) |
| **Vitamin supplement** |  |
| No | 5 (25.0) |
| Yes | 15 (75.0) |
| **Chemical substances exposure** |  |
| No | 12 (60.0) |
| Yes | 1 (5.0) |
| I don't know | 7 (35.0) |
| **Infections during pregnancy ‡** |  |
| No | 15/19 (78.9) |
| Yes | 4/19 (20.9) |
| **Urinary infections** |  |
| No | 16/19 (84.2) |
| Yes | 3/19 (15.8) |
| **Genital infections** |  |
| No | 18 (90.0) |
| Yes | 2 (10.0) |
| **Artificial Reproductive Technology use** | |
| No | 19 (95.0) |
| Yes | 1 (5.0) |
| **Previous pregnancies** |  |
| 0 | 7 (35.0) |
| 1 | 6 (30.0) |
| 2 | 5 (25.0) |
| >2 | 2 (10.0) |
| **Previous vaginal deliveries *** |  |
| 0 | 8/15 (53.0) |
| 1 | 6/15 (40.0) |
| 2 | 1/15 (6.7) |
| **Previous cesarean deliveries *** |  |
| 0 | 12/15 (80.0) |
| 1 | 2/15 (13.3) |
| 2 | 1/15 (6.7) |
| **Number of stillbirths *** |  |
| 0 | 13/15 (86.7) |
| 1 | 2/15 (13.3) |
| **Number of previous miscarriages**§ |  |
| 0 | 4/14 (28.6) |
| 1 | 9/14 (64.3) |
| 2 | 1/14 (7.1) |

* Information available for 15/20 women. ‡ Information available for 17/20 women. § Information available for 14/20 women.
